# Whey Proteins and Bioactive Peptides: Advances in Production, Selection and Bioactivity Profiling

**DOI:** 10.3390/biomedicines13061311

**Published:** 2025-05-27

**Authors:** Anna Luparelli, Daniela Trisciuzzi, William Matteo Schirinzi, Leonardo Caputo, Leonardo Smiriglia, Laura Quintieri, Orazio Nicolotti, Linda Monaci

**Affiliations:** 1 Institute of Sciences of Food Production, National Research Council (ISPA-CNR), Via G. Amendola, 122/O, 70126 Bari, Italy; annaluparelli@cnr.it (A.L.); williamschirinzi99@gmail.com (W.M.S.); leonardo.caputo@cnr.it (L.C.); 2 Department of Pharmacy, Pharmaceutical Sciences, University of Bari “Aldo Moro”, Via E. Orabona, 4, 70125 Bari, Italy; daniela.trisciuzzi@uniba.it; 3 Parafarmacia Smiriglia Leonardo, Via S. Giorgio, 19/B, Triggiano, 70019 Bari, Italy; smiriglialeonardo@libero.it; 4 Institute of Biomembranes, Bioenergetics and Molecular Biotechnologies, National Research Council (IBIOM-CNR), Via G. Amendola, 122/O, 70126 Bari, Italy; linda.monaci@cnr.it

**Keywords:** whey proteins, β-lactoglobulin, α-lactoalbumin, minor species, bioactive peptides, hydrolysis, machine learning, biological prediction, in silico methods, WP-derived BAPs legislation

## Abstract

The whey protein (WP) fraction represents 18–20% of the total milk nitrogen content. It was originally considered a dairy industry waste, but upon its chemical characterization, it was found to be a precious source of bioactive components, growing in popularity as nutritional and functional food ingredients. This has generated a remarkable increase in interest in applications in the different sectors of nutrition, food industry, and pharmaceutics. WPs comprise immunoglobulins and proteins rich in branched and essential amino acids, and peptides endowed with several biological activities (antimicrobial, antihypertensive, antithrombotic, anticancer, antioxidant, opioid, immunomodulatory, and gut microbiota regulation) and technological properties (gelling, water binding, emulsification, and foaming ability). Currently, various process technologies and biotechnological methods are available to recover WPs and convert them into BioActive Peptides (BAPs) for commercial use. Additionally, in silico approaches could have a significant impact on the development of novel foods and/or ingredients and therapeutic agents. This review provides an overview of current and emerging methods for the production, selection, and application of whey peptides, offering insights into bioactivity profiling and potential therapeutic targets. Recent updates in legislation related to commercialized WPs-based products are also presented.

## 1. Introduction

Whey is a byproduct of dairy industries, which is formed during the milk coagulation process in the manufacture of dairy products. Considering that approximately 9 kg of whey is produced for 1 kg of cheese, a production of 187–206 million tons of whey was reported for 2023, and 203–241 million tons is estimated by 2030 [1]. In the past, whey was considered a waste, and there was no sustainable management of its disposal, which consisted of discharge into wastewater, into water without pretreatment or distributed onto the land [2]. This procedure is highly risky for the environment due to the high polluting power of whey linked to the high Biological and Chemical Oxygen Demand (BOD and COD) of 20–60 g/L and 50–102 g/L, respectively [3]. Although approximately 40% of whey is still discarded globally [4], the implementation of waste regulations and greater attention to the environment have encouraged the valorization of whey in a circular economy perspective.

Whey represents, in fact, a very rich source of nutrients, such as soluble proteins, lactose, vitamins, minerals, and fat [5,6], endowed with nutritional [1,7,8], techno-functional [9,10], and bioactive properties [3,11,12,13,14,15].

Whey proteins (WPs) constitute around 20% of the total protein content in milk [16] and include β-lactoglobulin (β-LG), α-lactalbumin (α-LA), immunoglobulins (IGs), bovine serum albumin (BSA), lactoferrin (LF), and lactoperoxidase (LP), together with other minor components [17]. Except for some minor species, the nutrient contents in whey from several mammal species are quite similar [18]. In general, WPs from alternative milk sources (e.g., buffalo, sheep, goat, camel, horse, yak, and donkey) are less commonly utilized, mainly due to limited awareness and availability; however, increasing interest in their properties and their potential application in dairy industries has recently been documented [19,20,21].

WPs are renowned for their high-quality protein content and excellent amino acid profile, which ensures good digestibility and provides essential amino acids that are readily absorbed and metabolized by the body [4,9]. For instance, sulfur amino acids play a role as antioxidants and as precursors of the powerful intracellular antioxidant glutathione [22]; amino acids such as isoleucine, leucine, and valine are critical as regulators of various metabolic functions and blood glucose homeostasis [8,22]. A recent narrative review explored the health implications of WPs supplementation, highlighting both benefits and potential risks [23]. WP dietary supplementation is avoided in subjects with hepatic and renal compromised functions, as well as in acne susceptibility; beneficial effects can be found in the intestinal microbiota, emotional and behavioral responses, and bone and muscle mass in elderly [23]. This study underscores the need for balanced WP consumption and further research to clarify its long-term health impacts, urging health professionals and consumers to make informed decisions [23].

Both WPs and their peptides have been widely studied for their ability to elicit positive physiological effects, suggesting food and pharmaceutical applications [5,15].

BioActive Peptides (BAPs) are generally released upon enzymatic breakdown during gastrointestinal digestion by proteinases (e.g., pepsin, chymotrypsin, and trypsin) and peptidases (e.g., carboxypeptidase, enteropeptidase, aminopeptidase, and dipeptidyl-peptidase IV) or during food processes. BAPs from whey proteins exhibit various biological activities, including antimicrobial, antihypertensive, antithrombotic, anticancer, antioxidant, opioid, immunomodulatory, and gut microbiota regulation [15].

In general, WPs are more rapidly digested than caseins in the gastrointestinal tract (2 vs. 7 h, respectively; [17]); however, excessive heating can potentially reduce WPs digestibility by modifying the structure of native proteins, which in turn limit cleavage site accessibility to enzymes [24]. Moderate heating during processing typically has a minimal impact on WPs’ gastroenteric digestibility [25,26].

**Figure 1:**
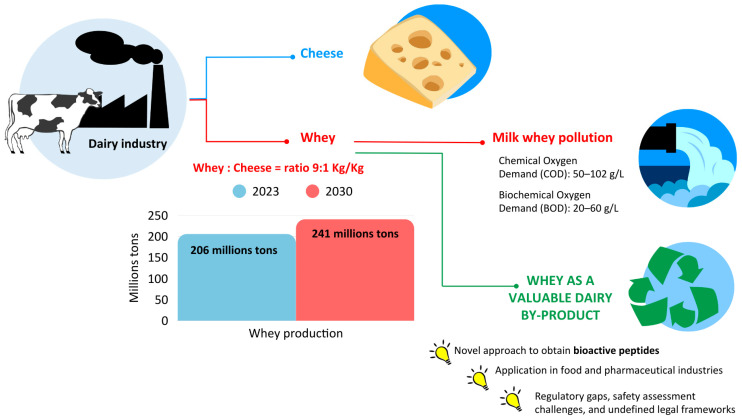
Whey: from waste to precious resource. The data regarding whey production and pollution were reported by Buchanan et al. [1].

In the dairy industry, milk processing promotes the release of BAPs of different molecular weights [27]; various proteinases are naturally present in milk, generally classified as cysteine- (cathepsin B), serine- (plasmin), metalloproteinase- (thermolysin), and aspartic-proteinase (cathepsin D). Among these proteinases, the most important is surely plasmin, the active form of plasminogen released from casein micelles following their destabilization under appropriate pH, temperature, and ionic conditions [28,29]. Besides rennet enzymes (chimosin, pepsin), non-dairy related proteases, including plant cardosin, bromelain, papain, actinidain, ficin, fungal flavorzyme, and bacterial subtilisin, have also been used to obtain functional foods [30,31] or to produce hydrolysates enriched in antimicrobial and antioxidant peptides; the latter have been successfully exploited both to extend the shelf-life and enhance the safety of refrigerated minimally processed foods [12,32,33,34,35]. Moreover, flavorzyme produced by *Aspergillus oryzae* is used to enhance flavor in various food products by hydrolyzing proteins, releasing amino acids that contribute to savory tastes, and debittering [36]. Figure 1 provides an overview of the impact of whey recovery and its transformation into high-value products.

Current trends focus on the mass production of BAPs. Besides conventional protocols, novel green technologies (e.g., ultrasound, microwave, hydrostatic pressure, pulsed electric field, and subcritical water) have made significant progress and have been applied to improve the yield and peptide stability; innovative membrane reactor for enzymatic hydrolysis, immobilized enzymes, and genetic and combinatorial approaches are also being developed for increased hydrolysis rates and greater production [37,38,39]. More targeted approaches have recently been described for the release of specific BAPs in a more predictable and efficient manner. These targeted approaches are mostly based on in silico QSAR models, molecular docking, and molecular dynamics simulation; taken together, these methods can significantly accelerate the identification of new BAPs and improve bioactivity and pharmaceutical properties [40]. Although the peptide therapeutics market is expected to expand over the coming years [41], the list of commercial products containing BAPs is still limited [15,42], and further research to transfer BAPs from laboratory- to large-scale production is still needed. One possible reason for this limitation is that the safety assessment of BAPs is still under investigation [15,21,43]. Chalamaiah et al. [44] recently discussed the lack of scientific validation of the safety and efficacy of many products already on the market that are based on BAPs, including those derived from WPs. This is frequently in contrast to the strict regulations on health claims that are in place in many countries. It is imperative that this scientific gap be addressed as soon as possible by validating precise clinical investigations, evaluating their safety, and determining the intended customer base for goods containing BAPs.

In this fascinating scenario, this review primarily focuses on the description of major and minor WPs and their related BAPs, highlighting the benefits associated with their applications in the food and pharmaceutical sectors. A comprehensive analysis of emerging trends in the production of BAPs from WPs is presented, with an emphasis on innovative extraction methods that enhance yield, efficiency, and functionality compared to conventional techniques.

Additionally, this study delves into the advanced exploration of novel and breakthrough techniques, predicting their biological profile. By leveraging state-of-the-art technologies and computational approaches, this study offers a comprehensive perspective on the potential of whey-derived BAPs to advance nutrition, drive innovation in functional foods, and support therapeutic applications. Notably, it also addresses the regulatory frameworks, existing gaps, and safety assessment challenges related to these compounds; the latter aspect is still unexplored in the current scientific literature.

## 2. Major and Minor WPs

WPs include five major protein fractions: beta-Lactoglobulin (β-LG), alpha-Lactalbumin (α-LA), bovine serum albumin (BSA), immunoglobulins (IGs), and Glycomacropeptide (GMP). In addition, minor protein fractions such as lactoferrin (LF), lactoperoxidase (LP), and lysozyme (LYZ) contribute to its overall composition. A positive impact on human health linked to the consumption of WPs has been widely reported [5,15,21,23,45,46,47,48].

Bovine WPs have been extensively utilized as a substrate for peptide release. Thus, recent advances in the field include WPs from minor species [21,49,50,51]. Table 1 compares minor and major dairy mammal species in terms of their WP composition: minor species like horse (7.4–9.1 g/L) and donkey (4.9–8 g/L) milk have a relatively high whey protein content, comparable to major species like sheep (10.2–11 g/L) but higher than cow (5.5–7 g/L) and goat (3.7–7 g/L) milk. This makes minor species’ milk a potential alternative for protein-rich diets. Camel milk lacks β-LG, while other species, such as buffalo (3.9 g/L) and yak (3.4–10.1 g/L), have significant levels. β-LG is crucial in milk allergy studies, and camel milk’s absence of this protein is a unique feature [52]. Compared with bovine milk, equine milk contains less β-LG and more α-LA and IGs.

The principal anti-microbial agent in milk is LYZ, and, to a lesser extent, LF, which predominates in horse, donkey, yak, and camel milk, which can contain up to 7.28 g/L [20,51,52]. Both LF and LYZ are very low in bovine milk, in which IGs form the main defense against microbes [53]. Together, IgA, IgG, IgM, LF, and LYZ provide the neonate with immune and non-immune protection against infection [54,55].

Minor dairy mammals, such as donkeys and camels, are notable for their unique protein compositions. Donkey milk, for instance, is exceptionally rich in LYZ, a protein with strong antimicrobial properties, while camel milk stands out for its high LF content, which is known for its antimicrobial and anti-inflammatory benefits [56,57]. These characteristics make milk from minor species particularly appealing to hypoallergenic and therapeutic applications, offering promising potential in specialized nutrition. On the other hand, major dairy mammals like sheep and buffalo provide milk with a rich protein profile, especially in WPs and immunoglobulins [58]. This makes their milk an excellent choice for developing high-protein dairy products, catering to the nutritional needs of a wide range of consumers. Together, these differences highlight the unique advantages of both minor and major dairy species in supporting human health and nutrition.

For further information, a brief description of each WP related to molecular structure and healthy effects is reported below.

**Table 1:** Content of WPs in whey from major and minor mammal species.

Proteins (g/L)	Minor Dairy Mammal Species				Major Dairy Mammal Species				References
	**Horse**	**Donkey**	**Camel**	**Yak**	**Cow**	**Goat**	**Sheep**	**Buffalo**	[20,51,57,58]
Total WPs	7.4–9.1	4.9–8	5.9–8.1	10	5.5–7	3.7–7	10.2–11	6	
β-lactoglobulin (β-LG)	2.55	3.3	N/A *	3.4–10.1	3.2–3.3	1.5–5.0	6.5–8.5	3.9	
α-lactoalbumin (α-LA)	2.37	1.9	0.8–3.5	0.2–1.7	1.2–1.3	0.7–2.3	1–1.9	1.4	
Serum Albumin	0.37	0.4	7–11.9	0.2–3.1	0.3–0.4	0.25–1.1	0.4–0.6	0.29	
Immunoglobulins (IGs)	1.63	1.30	1.5–19.6	0.1–0.4	0.5–1.0	0.1–0.5	0.7	10.66	
IgG	0.38	N/A *	0.72–2.23	N/A *	0.15–0.8	0.1–0.4	N/A *	0.37–1.34	
IgA	0.96	N/A *	N/A *	N/A *	0.05–0.14	0.03–0.08	N/A *	0.01–0.04	
IgM	0.02	N/A *	N/A *	N/A *	0.04–0.1	0.01–0.04	N/A *	0.04–1.91	
Lactoferrin (LF)	0.1–2.0	0.07–0.37	0.02–7.28	0.1–0.7	0.02–0.5	0.02–0.2	0.8	0.03–3.4	
Lysozyme (LYZ)	0.5–1.33	1.00–1.43	0.000060–0.00135	N/A *	0.000070–0.00060	0.000250	0.000100	0.000120–0.000152	
Proteose–peptone (Pp)	N/A *	N/A *	N/A *	N/A *	0.8–1.2	N/A *	N/A *	3.31	

* N/A (not available).

### 2.1. β-Lactoglobulin (β-LG)

β-LG is the major WP in the milk of almost all mammalian species, including cow, sheep, goat, donkey, and mare [59]; β-LG is a water-soluble globular protein that constitutes approximately 50% of total whey proteins, with a chain of 162 amino acids and a molecular weight of around 18.4 kDa [59]. This protein is an important source of cysteine, which is fundamental for the synthesis of glutathione (GSH), a well-known intracellular antioxidant involved in immune regulation, cancer prevention, and the improvement in liver function [60]. Beyond their well-documented nutritional and functional roles, the structural characteristics of globular whey proteins, such as β-lactoglobulin, also possess antioxidant properties that are increasingly relevant in food science. These proteins can act as antioxidants and free radical scavengers, a property linked to their structural characteristics, including disulfide bonds and hydrophobic interactions. Such antioxidant activity contributes to the oxidative stability of food products, thereby extending shelf life and enhancing quality. In this regard, incorporating these WPs in formulations could therefore serve a dual role, offering both functional performance and bioactive protection.

Furthermore, one of the most important physiological functions is the stimulation of lipase activity [59]. β-LG is also an excellent source of essential amino acids such as leucine, which triggers the anabolic properties of muscles, leading to a reduction in the risk of sarcopenia in older adults [61]. β-LG may also be involved in fatty acid metabolism due to a ligand-binding site that tends to bind to hydrophobic molecules such as fat-soluble vitamins as well as lipids [62]. All these aspects make this protein an attractive compound for the formulation of nutraceutical foods and can help to avoid diseases and health issues.

Dairy industries have shown increasing interest in the reuse of whey due to its techno-functional properties bestowed mostly by β-LG, such as the ability to form gels and aggregates and the possibility of using this protein as an emulsifier in various food formulations [63]. A recent study highlights the successful gelation of β-LG, a whey protein, using heat treatment and transglutaminase-catalyzed crosslinking. Optimized conditions (pH 7.5, 5% protein concentration, 80 °C for 30 min) yielded soft, nutritious gels ideal for texture-modified diets, particularly for individuals with dysphagia or the elderly [64]. These gels offer an alternative to agar, improving dietary management and quality of life.

However, β-LG is an allergenic protein in bovine milk intended for human infants. It is known that β-LG increases in antigenicity and allergenicity when subjected to temperatures ranging from 50 to 90 °C; on the contrary, a decrease was observed after 90 °C [65]. For this reason, the development of foods with a reduced content of β-LG has generated much interest [66,67].

In addition to its usefulness in the food industry, the literature has reported interesting and innovative applications of β-LG in the pharmaceutical field, emerging as a promising excipient for amorphous solid dispersions to enhance the oral bioavailability of poorly water-soluble drugs [68].

### 2.2. α-LActoalbumin (α-LA)

α-LA is the second most abundant WP, accounting for approximately 20% of the total whey protein content. It consists of an amino acid chain with a molecular weight of approximately 14.4 kDa [69].

α-LA contains a high percentage of essential amino acids (63.2% of the total amino acid content) [69], more specifically, lysine, cysteine, and tryptophan [70]. Due to its high tryptophan content as a precursor to serotonin or melatonin, α-LA can help improve mood, sleep, and congestive performance [71,72]. This protein has also been shown to possess anti-tumor activity, as it can selectively induce apoptosis in tumor cells [73]. Additionally, α-LA can form a complex with oleic acid at low pH values, imparting anticancer properties [73].

Human and bovine α-LA share similar amino acid compositions, having 74% sequence homology and similar bioactivities; in fact, it is mostly used in infant formula [74]. It is also involved in the regulation of lactose synthesis, contributing to lactose synthetase, the enzyme that catalyses the final step in the biosynthesis of lactose [75]. α-LA is an important transporter of calcium (Ca^2+^) and can bind two molecules, thanks to a pocket containing four aspartic acid (Asp) residues. However, at pH < 5, Asp becomes protonated and loses its ability to bind Ca^2+^. The calcium-containing protein denatures reversibly at relatively low temperatures but renatures in the absence of other WPs and is therefore considered the most heat-stable of the major WPs [66]. In camel whey, α-LA is the major WP of camel milk because of the absence of β-LG; camel α-LA presents differences in its structural characteristics compared with its bovine counterpart, leading to diverse therapeutic properties, biological activities, and techno-functional properties [59,76].

Among the applications, Lu et al. [77] synthesized a dihydromyricetin/α-LA covalent complex with strong antioxidant and glucosidase inhibitory activities. The authors proposed its use as an emulsifier to stabilize β-carotene-loaded nano-emulsions, highlighting its potential for innovative food delivery systems.

α-LA has a wide range of potential applications in food technology, pharmaceutics, and nanotechnology. As a source of BAPs with anticancer, antimicrobial, antiviral, antihypertensive, immunomodulating, opioid, mineral-binding, and antioxidant properties, it plays a key role in the formulation of functional foods. Additionally, nanotubes and nanoparticles derived from this protein serve as effective carriers for transporting active compounds, positioning α-LA as a valuable tool for both the food and pharmaceutical industries [69].

### 2.3. Albumin of the Bovine Serum (BSA)

BSA amounts to approximately 8% of total WPs, with a chain of 582 amino acids and a molecular weight of 69 kDa. BSA is not synthesized in the mammary gland but appears instead in milk following passive leakage from the bloodstream [66]. Because of its size and higher levels of structure, BSA can bind to free fatty acids and other lipids, as well as flavor compounds [78]. Very recently, BSA has been used as a promising anticancer therapeutic strategy: using *Escherichia coli* surface-modified by BSA resulted in stronger adhesion and targeting effects on bladder cancer cells, paving the way for future studies exploring the combination of BSA combined with live bacteria for cancer therapy [79]. Additionally, BSA is a precursor for the synthesis of glutathione, a peptide associated with the immunostimulatory effect of whey proteins [80].

The effect of BSA or whey protein isolate (WPI) at various concentrations (0.5%, 1.5%, and 3%; *w/v*) on the properties of shrimp oil-in-water emulsion was investigated in a recent study [81]. BSA–Chitosan-stabilized emulsions showed greater stability, with a smaller droplet size enlargement during storage and shear-thinning, non-Newtonian behavior. They also optimally retained prawn oil pigment stability when compared to WPI– Chitosan-stabilized emulsions [81].

In a recent study, whey protein–polysaccharide nanotubes, such as α-LA–chitosan and bovine serum albumin–κ-carrageenan ones, were used to encapsulate curcumin [82]. It was reported that the encapsulation efficiency of curcumin ranged between 40% and 45%, and the curcumin-loaded nanotubes exhibited some anticancer effects when using a cell culture model.

### 2.4. Immunoglobulins (IGs)

IGs exert important immunological protection for neonates, combat infections, and enhance gut health. IGs are globular glycosylated proteins with a molecular weight between 22 and 69 kDa and various forms and structures. They amount to around 10% of total WPs, and their content is mainly high in colostrum and drops significantly during lactation. Horse, buffalo, and camel whey contain higher IG levels compared to bovine, sheep, goat, and human milk, with variations in Ig ratios across species [83]. IgG predominates in horse colostrum, as well as in bovine milk and colostrum, whereas IgA is the major immunoglobulin in mature horse milk and human milk and colostrum [84]. Immune milk was also suggested to lower blood pressure [85].

In a double-blind clinical study, the effects on the reduction in plasma cholesterol and blood pressure were evaluated by involving hypercholesterolemic human subjects. Patients consumed 90 g per day of immune milk produced from dairy cows previously hyperimmunized with a multivalent bacterial vaccine, which was found to be a useful adjuvant in the dietary management of hypercholesterolemia [85].

Furthermore, several studies have shown that milk IGs are effective against rotavirus and other infections [86].

### 2.5. Glycomacropeptide (GMP)

GMP, also called caseinomacropeptide, is a phosphorylated and glycosylated milk-derived bioactive peptide that is released from κ-casein via enzymatic digestion, either physiologically or in industry during the cheese-making process. GMP (12%) is characterized by an imbalanced amino acid profile. It lacks essential aromatic amino acids such as tryptophan, phenylalanine, and tyrosine, as well as cysteine, but is notably rich in threonine, proline, glutamine, and serine [87].

In addition to its nutritional value, GMP has demonstrated beneficial effects on satiety and the management of phenylketonuria [88]. It exhibits several other bioactive functions that may have therapeutic potential, including the modulation of digestion and metabolism and antibacterial, prebiotic, remineralizing, anti-tumoral, and immuno-modulation activities. In vitro and in vivo evidence suggests the activity may at least in part be associated with post-translational glycosylation of the κ-casein protein [88].

Regarding antibacterial activity, GMP has demonstrated significant inhibitory effects on the adhesion of pathogenic bacteria to target cells, mediated by interactions involving sialic acid or other carbohydrate structures [89,90]. The inhibitory effect of GMP was observed in the adhesion of verotoxigenic and enteropathogenic *E. coli* to HT-29 human colon adenocarcinoma epithelial cells [91]; *E. coli*, *S. typhimurium*, and *Shigella flexneri* to Caco-2 cells [92]; and enterotoxigenic *E. coli K88* to piglet ileal mucosa cells [90]. Notably, this activity was enhanced when GMP underwent protease digestion, and it was demonstrated that GMP interacts directly with the bacteria rather than with their target cells [90]. While substantial in vitro and in vivo evidence supports GMP’s protective effects against enteropathogenic bacterial infections, further research is needed to clarify its mechanisms of action and rule out potential dysbiosis. GMP has shown remineralizing effects, promoted mineral recovery in demineralized bovine enamel [93], improved zinc absorption in infant rhesus monkeys [94], and mitigated bone damage in mice [95]. In male mice on a low-calcium diet, GMP increased the femoral calcium concentration, particularly during growth phases [96], while in female mice, GMP supplementation enhanced bone quality under both high- and low-fat diets [97]. These benefits may be linked to GMP’s prebiotic activity and its role in increasing the cecal concentration of short-chain fatty acids, though further research is needed to confirm this mechanism.

Furthermore, GMP may have a role as a prebiotic in promoting the growth of healthy gut microbiota, particularly *Bifidobacterium* and *lactic acid bacteria*, which may prevent or attenuate the growth of pathogenic bacteria [98].

GMP shows promise in combating metabolic syndrome by targeting key risk factors such as inflammation, oxidative stress, and endoplasmic reticulum stress, independent of intestinal microbiota. In high-fat, high-fructose-diet-fed mice, GMP administration for 12 weeks reduced obesity, hyperglycemia, and hyperinsulinemia, improving insulin resistance [99]. Additionally, in the same study, GMP decreased systemic inflammation, enhanced insulin sensitivity, and restored intestinal and hepatic homeostasis by mitigating diet-induced tissue stress and inflammation. Studies on the immunomodulatory activity of GMP, including cellular, animal, and human trials, suggest that GMP primarily downregulates inflammatory pathways, reducing cytokines like TNF-α. However, occasional up-regulation of inflammatory markers has also been observed, indicating a context-dependent response [100].

### 2.6. Lactoferrin (LF)

LF is an iron-binding glycoprotein, with a MW of 80 kDa, and amounts to around 1–3% of total WPs [26]. LF exhibits multiple biological functions, such as enhanced iron bioavailability, antimicrobial and antibiofilm properties, antioxidant, antiviral, anti-inflammatory, immunomodulatory, and anticancer effects [12,13,101]. Owing to its diverse benefits, bovine LF is extensively employed in both human medicine and the food industry [101]. Compared to bovine milk, camel whey has higher concentrations of LF, which exhibit notable antibacterial, antiviral properties, hypoglycemic, antidiabetic, anti-inflammatory, and immunomodulatory effects [102,103].

Extensive literature and animal model studies highlight the antimicrobial efficacy of LF. This activity is mediated through two mechanisms: the sequestration of free iron, which inhibits bacterial growth by depriving bacteria of an essential nutrient (bacteriostatic effect), and direct interaction with pathogens. LF binds to bacterial wall lipopolysaccharides and may cause membrane damage through peroxide formation catalyzed by LF-bound iron, ultimately leading to bacterial cell lysis [104].

It can protect the mammary gland and gastrointestinal tract against infections; provides and is a rich source of amino acids; and promotes division, differentiation, and growth of the immune system and mucosa intestinal cells. Human milk contains a very high level of LF (~20% of the total nitrogen), and therefore, there is interest in fortifying bovine milk-based infant formulas with this protein [66]. LF is widely incorporated into a variety of foods, including probiotic foods that promote beneficial gut flora and functional foods designed to enhance iron absorption [105,106]. Bovine LF supplementation has demonstrated significant positive effects across various preclinical and clinical models, improving not only the composition of the gut microbiota but also crucial physiological responses such as inflammation, cognitive health, and therapy response.

In mouse models of colitis induced by dextran sulfate sodium salt, oral supplementation with bovine LF (100 mg/kg) reduced inflammation and improved the structure of the colon barrier, demonstrating how LF can alleviate colitis symptoms by modulating the gut microbiota [107]. Additionally, in high-fat diet-induced obese mice, supplementation with bovine lactoferrin resulted in a significant alteration of the microbiota, increasing *Bifidobacterium* and decreasing *Enterobacteriales* and *Bacteroidetes*. These effects were accompanied by reductions in cholesterol and glucose levels, suggesting an improvement in diet-induced intestinal dysbiosis [108]. In the case of C57BL/6J mice fed a Western diet, lactoferrin supplementation (50 mg/kg BW) for 16 weeks led to an increase in Bacteroidetes (e.g., *Roseburia*), suggesting a potential beneficial effect on the gut–brain microbiota axis with possible implications for cognitive health [109]. In weaning piglets (1–3 g/kg), lactoferrin supplementation enhanced growth performance and reduced diarrhea rates by improving gut barrier function and balancing the intestinal microbiota, increasing *Lactobacillus* and *Bifidobacterium* [110].

In pediatric patients undergoing chemotherapy, supplementation with bovine lactoferrin (200 mg/day) for two months promoted gut microbiota eubiosis by controlling the growth of pathobionts such as *Enterococcus* and modulating the abundance of beneficial taxa like *Akkermansia*. This approach helped counteract dysbiosis onset, improving overall health and therapy response [111]. Finally, in healthy elderly women, lactoferrin supplementation (1 g/day), alone or in combination with galacto-oligosaccharides and vitamin D, increased *Holdemanella* and *Bifidobacterium* in the fecal microbiota, though health effects have yet to be assessed [112].

Additionally, LF plays a role in food preservation by delaying lipid oxidation and inhibiting microbial growth. Its use spans industries including meat, wine, fat processing, and dairy [42]. The increasing demand for natural foods has further elevated the importance of natural inhibitors like LF. It is also used in skin care products for its antioxidant properties [113], in oral care products for oral hygiene [114], and as a nutraceutical to boost the immune system and reduce inflammation [115].

### 2.7. Proteose–Peptone (Pp)

Pp, also called lactophorin, is a milk protein fraction known for its heat stability and acid solubility, and it is a mixture of phospho- and glycoprotein and peptides. It is a heat-stable and acid-soluble protein fraction in milk. Pp was indigenous in fresh milk and does not result from heat-induced hydrolysis of the major milk proteins [116]. It is obtained by subjecting milk to heat treatment and adjusting its pH to 4.6, which allows its separation from other milk proteins. Pp has been shown to act as an effective emulsifier in oil-in-water emulsion systems by rapidly reducing interfacial tension. This property makes Pp a functional ingredient in products like ice cream, which rely on stable emulsions or foams [117]. A recent study highlights the species-specific bioactive properties of Pp fractions and their potential applications [118].

This study explored the bioactivity of bovine and ovine proteose–peptone fractions, focusing on proteose–peptone component 3 (Pp3) and its enzymatic hydrolysate, with particular attention to antioxidant, antibacterial, and antiviral properties. Antioxidant activity differed significantly between species: bovine Pp exhibited a reduction after fractionation, while ovine Pp showed an increase, consistently displaying superior activity.

Regarding antibacterial effects, hydrolyzed bovine Pp3 demonstrated a strong, concentration-dependent action against *Cronobacter sakazakii*, achieving a maximum reduction of 6.82 logarithmic cycles at 4 mg/mL after 24 h, whereas ovine Pp3, whether intact or hydrolyzed, was ineffective. In terms of antiviral activity, both bovine and ovine Pp3 neutralized rotavirus in a dose-dependent manner; however, hydrolysis enhanced the antiviral properties of bovine Pp3 while nearly eliminating the activity of ovine Pp3. These findings underscore significant interspecies variations in the bioactivity of Pp fractions [118].

### 2.8. Lactoperoxidase (LP)

LP accounts for 0.25–0.5% of whey proteins and has a molecular weight of 78 kDa [119]. It consists of a single polypeptide chain with 612 amino acid residues, including 15 cysteines, one heme group, and approximately 10% *w*/*w* carbohydrate moieties. The enzyme’s activity is temperature-dependent, with inactivation occurring at 62.5 °C after 30 min, 70 °C after 15 min, or 85 °C after 15 s [120,121]. LP exhibits broad antibacterial activity, making it a promising natural preservative for various food products [122,123]. However, its direct application can be limited due to its diffusion within the food matrix and interactions with food components. To overcome these challenges, LP can be incorporated into packaging materials, particularly edible films and coatings, to retain its antimicrobial properties, thereby enhancing food safety and extending shelf life. This study reviews antimicrobial enzymes, emphasizing LP’s properties and its potential role in combination with edible films for food preservation [122]. Min et al. [124] evaluated the antimicrobial effects of WPs films containing LP against *Salmonella enterica* and *E. coli O157:H7*, achieving a 4 log CFU/cm^2^ reduction. Similarly, WPI films with LPOS inhibited *L. monocytogenes* on agar media by 4.2 log CFU/cm^2^. When applied as a coating on cold-smoked salmon, LP-whey protein coatings (40 mg LP/g) reduced *L. monocytogenes* and total aerobic microorganisms by approximately 3 and 1 log CFU/g, respectively, with inhibition maintained for 35 days at 4 °C and 14 days at 10 °C. The reduced LP efficiency in smoked salmon, compared to agar media, was linked to its higher SH compound content and lower water activity, which hinders the diffusion of oxidizing agents [124].

Saravani et al. [125] evaluated whey protein coatings with LP and *Bunium persicum* essential oil on Gouda cheese and chicken fillets. The coatings stabilized bacterial counts in cheese and reduced lipid oxidation, with stronger antibacterial effects observed against Gram-negative bacteria. In chicken, coatings with LP and 1% essential oil significantly inhibited *L. monocytogenes*. LP’s antibacterial activity varies by bacterial type, often requiring combination with other preservation methods for broader efficacy.

### 2.9. Lysozyme (LYZ)

LYZ is a protein consisting of 129 amino acids and stabilized by four disulfide bonds, although only two of these are essential for its hydrolytic activity [120]. It has a molecular mass of 14,300–14,600 Da and an isoelectric point between 9.5 and 9.7. Similarly to LF, LYZ’s antibacterial properties contribute to simplifying the gut microbiota, boosting resistance to intestinal colonization by certain pathogens, while promoting the growth of beneficial bacteria and aiding recovery from various gastrointestinal disorders [126]. LYZ exhibits antimicrobial properties against *Salmonella typhimurium* and *Escherichia coli* [124]. However, its effectiveness against gram-negative bacteria is limited due to the presence of a lipopolysaccharide layer in their outer membrane, which acts as a barrier preventing LYZ from accessing the cell interior. Recently, the inclusion of antimicrobial enzymes, particularly LP and LYZ, in active packaging materials such as cellulose, gelatin, and chitosan edible coatings and films has emerged as a promising approach to preserve the enzymes’ catalytic activity and prevent food spoilage microorganisms [127,128]. Notably, a few antimicrobial enzymes, including LYZ and LP, are classified as GRAS (Generally Recognized As Safe) and have been approved for use in food products [129].

Major characteristics and biological effects above described are summarized in Table 2.

## 3. Conventional vs. Novel Approaches for the Release of BAPs from WPs

**Figure 2:**
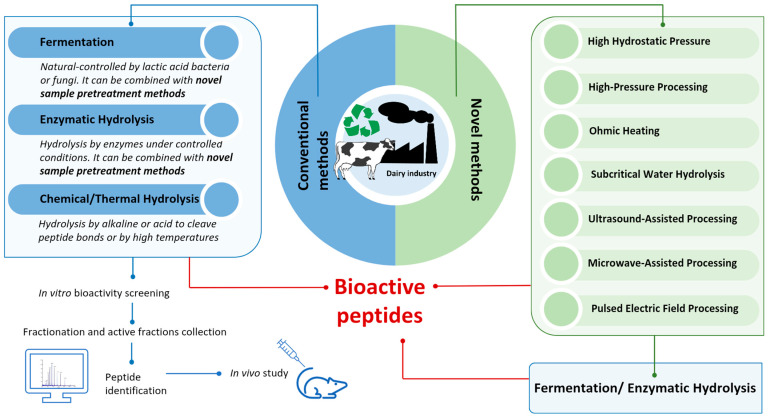
Classical and novel processing techniques to discover and obtain bioactive peptides (BAPs) from whey.

BAPs are specific protein fragments which, in addition to having a purely nutritional value, exert biological activity on the organism. Being often latent or encrypted within parent proteins, they can be released through enzymatic actions during gastrointestinal digestion or food processing. The size of BAPs is usually 2–20 amino acids long, and their primary sequence defines their function [134]. To date, the development of novel technologies—coupled or not with conventional methods and able to generate high yields of BAPs from WPs quickly and at a low cost—is ongoing [135]. Emerging processing technologies, such as ultrasound-assisted processing, microwave-assisted processing, high-pressure processing, pulsed electric field processing, high hydrostatic pressure, subcritical water hydrolysis, and ohmic heating, utilize physical methods to enhance the degree of hydrolysis in the production of BAPs, as reported in Figure 2 [135,136,137]. These novel techniques are increasingly used in the dairy industry to prepare BAPs, offering potential improvements in efficiency and functionality. Although generally less efficient as standalone methods compared to traditional techniques, these novel approaches are often coupled with fermentation or enzymatic hydrolysis to enhance peptide production and improve the release of bioactive compounds [138,139,140].

Classical approaches (microbial fermentation, enzymatic hydrolysis, and thermal and chemical hydrolysis) and recent advances in this field (Figure 2) will be discussed in the following paragraphs. Advantages and disadvantages in industrial application are also summarized in Table 3.

### 3.1. Microbial Fermentation (MF)

MF is a widely used, cost-effective, and efficient technique for producing peptides (Table 3) [141]. This method plays a crucial role in the dairy industry, particularly in enhancing the functional properties of milk products and byproducts [141,142]. MF is generally more cost-effective than enzymatic methods for producing BAPs. However, it presents certain industrial limitations, such as low peptide yields and a lack of specificity in peptide generation [143]. On the other hand, using commercial enzymes to apply them in BAPs production processes could become a serious problem, since the enzyme market is already under an oligopoly [144]. Therefore, solid-state fermentation (SSF) based on the use of selected microorganisms (fungi and bacteria) capable of biosynthesizing mixtures of biopeptides even starting from food residues is becoming increasingly popular because of its ability to reduce environmental pollution, adding value to these residues and lower costs in the process. SSF also allows the simultaneous production of proteases and BAPs, optimizing the process and reducing the compounds [145]. Therefore, SSF could become a preferred route to develop BAP-enriched ingredients or foods without resorting to laborious extraction and purification procedures.

SSF has been widely used to produce enzymes and other bio compounds due to advantages such as simplicity, the possibility of using residues as substrates, and enabling a reduction in environmental pollution, adding value to these residues and lower costs in the process. SSF also allows the simultaneous production of proteases and BAPs, optimizing the process and reducing antinutritional compounds, such as allergens. This work aimed to review the literature on the production of proteases and BAPs by solid-state fermentation, including the simultaneous production of these compounds, since the fermented medium can benefit from the hydrolysis capacity of enzymes produced by microorganisms. Usually, the obtaining of these compounds has been studied in isolation and not simultaneously in a single bioprocess, this being the main difference of our review.

Lactic acid bacteria (LABs) and yeast strains are frequently utilized for whey fermentation due to their robust proteolytic activity and capacity to thrive in acidic environments [146]. LAB cell-envelope proteinases hydrolyze whey proteins to produce extracellular oligopeptides with potential bioactivity. These oligopeptides are subsequently transported into LAB cells via active transporters and further broken down into smaller peptides and free amino acids by intracellular endopeptidases [147].

Daliri et al. [142] demonstrated that various LAB families can hydrolyze WPs to generate BAPs with antihypertensive effects. After culturing 34 fermenting bacteria for 48 h at 37 °C, seven strains—including *Pediococcus acidilactici* SDL1414, *Lactobacillus plantarum* JDFM44, *Enterococcus faecium* SC54, *P. acidilactici* DM9, *Lactobacillus brevis* SDL1411, *Pediococcus pentosaceus* SDL1409, and *Lactobacillus rhamnosus* JDFM6—produced peptides (<7 kDa) with strong ACE-inhibitory activity.

*P. acidilactici* SDL1414, which exhibited the lowest half-maximal inhibitory concentration (IC50) value of 19.78 ± 1.73 μg/mL, produced 29 compounds with antihypertensive effects. These compounds were identified using Liquid Chromatography–Electrospray Ionization–Quantitative Time-of-Flight Tandem Mass Spectrometry (LC-ESI-TOF–MS/MS). This underscores the potential of *P. acidilactici* SDL1414 as a source of bioactive compounds with functional properties [142]. Antimicrobial peptides were released after the fermentation of goat whey with *Lactobacillus* spp. [148]; BAPs were also released from WP concentrate (WPC) with *Streptococcus thermophilus* [149], *Enterococcus faecalis* [150], *Saccharomyces* spp. [151], and natural whey microbial starters [152].

### 3.2. Enzymatic Hydrolysis

Besides fermentation, hydrolysis with purified proteases (or peptidases) is the most common method for producing BAPs [153]; however, despite their wide usage, current enzymatic treatments often face limitations such as low yields and high costs [153]. Many food-grade proteases from animal, plant, and microbial sources with high substrate specificity are already available on the market (Table 3) [154,155]; not surprisingly, the global hydrolyzed WP market was estimated at EUR 1.86 Billion in 2023 and is projected to reach EUR 3.0 Billion by 2030.

Kheroufi et al. [156] studied the biological (antioxidant) and technological properties (solubility, emulsifying activity index, and foaming activity) of WP hydrolysates (WPHs) obtained using chicken pepsin extract in comparison with porcine pepsin. Bromelain, papain, neutrase, and cardosin are other examples of enzymes usually used to obtain WPHs endowed with biological activities [157,158,159,160].

A recent study focused on hydrolysates from WPC, produced from Colombian cheese whey via ultrafiltration, followed by enzymatic hydrolysis with alcalase, chymotrypsin, and flavorzyme [161]; in particular, the alcalase hydrolysate exhibited higher ABTS radical scavenging capacity, while alcalase and chymotrypsin hydrolysates showed over 85% angiotensin-converting enzyme (ACE) inhibition. After simulated gastrointestinal digestion, changes in biological activities were observed: ABTS scavenging ability increased, while ACE inhibition significantly decreased. The alcalase hydrolysate demonstrated strong antioxidant and ACE-inhibitory properties and better peptide stability post-digestion [161]. Alcalase is known for its high efficiency in hydrolyzing various proteins to produce BAPs; additionally, immobilized alcalase has been proposed based on the affinity of different metal ions, enhancing its stability and catalytic activity for specific applications [162,163]. Immobilized alcalase can effectively hydrolyze whey protein isolate to produce small peptides (<500 Da). Immobilization improves the enzyme’s catalytic performance, particularly for proteins with low solubility. Alcalase@HMSS-3N–Mn+ prevents the enzyme from self-digestion and demonstrates excellent thermal stability, supporting the use of metal ion affinity chromatography for immobilizing enzymes and producing BAPs using various metal ions [162].

A promising method for generating BAPs involves the use of alcalase immobilized on glyoxyl-corn-cob-powder [163]. This method hydrolyzes cheese whey proteins while promoting sustainability by utilizing whey and corn cob, producing hydrolysates with strong bioactive properties suitable for food and pharmaceutical applications. Specifically, WP hydrolysates exhibited antioxidant activity, high iron-chelating capacity (76.2%), and antimicrobial effects (87.75–100%) against *E. coli* and *Listeria monocytogenes* and were effective against *Candida albicans* (MIC = 10 mg/mL) [163].

### 3.3. Chemical Hydrolysis and Heat Treatment

Chemical hydrolysis is a simple and cost-effective method for producing peptides and amino acids using acids or alkalis [164]. However, it suffers from issues like process control difficulties and variability in chemical compositions, as well as negative impacts on nutritional quality and functionality due to extreme temperature and pH conditions [165] (Table 3).

The chemical hydrolysis of WPs involves the disruption of sulfide bonds within protein structures, enhancing their function and properties. Lowering the pH with succinic acid or affecting disulfide bonds through sulfitolysis can improve protein properties [166]. For example, adjusting whey pH to 3.0–4.6 with citric acid yields high levels of soluble β-LG [167].

The heat treatment of WPs releases free thiol groups, causing denaturation and thiol-disulfide bond formation, with calcium aiding in thermal precipitation. Typically, dehydrated whey is denatured at 90 °C and precipitated at pH 4.4–5.0 for low-solubility protein with good functionality. The process involves acidification (pH 2.5–3.5) followed by heat. Iron addition improves solubility. Heat treatment of β-LG with lysine and varying calcium concentrations yielded seventeen peptides [168]. In addition, β-lactoglobulin denatured by thermal treatment (50–95 °C) provided the most effective protection against lipid oxidation in oil-in-water emulsions when used as a stabilizer [169].

### 3.4. Ultrasound Treatment

Ultrasound-assisted processing is an eco-friendly, non-thermal technology that employs ultrasonic waves (frequencies > 20 kHz) to enhance peptide production. The mechanical and cavitation effects generated by these waves efficiently modify protein structures, increasing enzymatic activity and accelerating hydrolysis processes [135]. A recent study highlighted that ultrasound treatment of WPI solutions is influenced by factors such as acoustic amplitude, treatment duration, and protein concentration [138]. Pretreatment with ultrasound before enzymatic hydrolysis using bromelain (a plant-derived protease) significantly modified the peptide profile of WPI hydrolysates. This process released peptides with enhanced ACE-inhibitory activity, particularly in the 1–5 kDa range, which notably remained stable after simulated gastric and intestinal digestion [138]. Ultrasound pretreatment enhances enzyme accessibility to peptide bonds, increasing the release of BAPs. This process generates acoustic forces that reduce the fat globule size, which are typically coated with whey proteins and casein micelles, thereby increasing the surface area available for proteolytic enzymes to interact with proteins. As a result, ultrasound improves enzymatic hydrolysis efficiency [170]. Wu et al. [171] demonstrated that ultrasonic pretreatment of WP prior to hydrolysis with alcalase significantly enhanced ACE-inhibitory and immunomodulatory activities. Ultrasound-induced protein unfolding increased free sulfhydryl content and promoted conformational changes, notably the formation of β-sheets and β-turns, which contributed to improved bioactivity of the hydrolysates [171]. Recent studies confirm the utility of ultrasound as a sample pretreatment technique, demonstrating its effectiveness not only in increasing the yield of BAPs from WPs but also in significantly reducing allergenicity [172]. This advancement highlights the potential of ultrasound-assisted methods for the development of hypoallergenic food products [173,174], offering promising applications in the food industry.

Studies have shown that ultrasound-assisted pretreatment, combined with low-purity enzymes, enhances the hydrolysis rate of proteins. This effect is attributed to structural modifications such as changes in free sulfhydryl groups, disulfide bonds, increased hydrophobic protein content, and surface hydrophobicity [42]. Lorenzetti et al. [139] further demonstrated that ultrasound pretreatment of WPI before hydrolysis could facilitate the development of cost-effective ingredients for the dairy industry, highlighting its economic and industrial relevance. Ultrasound-assisted processing systems are energy-efficient (up to 85%), require minimal maintenance, and involve moderate installation costs ranging from EUR 10,000 to EUR 200,000 [175]. Its numerous advantages, including faster processing, selective extraction, precise control, reduced temperature requirements, and efficient mass and energy transfer, make ultrasound-assisted processing a preferred technique for producing BAPs [176] (Table 3).

### 3.5. Microwave Treatment

Microwave-assisted processing employs electromagnetic waves with wavelengths ranging from 0.001 to 1 m and frequencies between 300 MHz and 300 GHz [177]. This technique enhances food shelf life and functional properties [135] while improving taste and overall quality [177]. Key parameters such as power, frequency, time, and temperature play a crucial role in the effectiveness of microwave-assisted processing [177].

Microwave energy induces molecular interactions through ionic conduction and dipolar rotation mechanisms, generating heat at the molecular level that leads to structural modifications in proteins [135]. Microwave-assisted extraction can operate through thermal or non-thermal mechanisms using nonionizing radiation. Thermal effects result from localized heat generated by water molecule friction, while nonthermal effects involve protein unfolding and rearrangement rates [135]. By breaking disulfide bonds, microwaves facilitate protein opening, enhancing enzymatic hydrolysis, like ultrasound-assisted extraction [177]. Specifically, applying a microwave-assisted protocol (532 W, 40–50 °C, 5 min) to bovine serum albumin concentrates prior to proteolysis with enzymes like pronase, chymotrypsin, papain, or alcalase has been shown to significantly increase hydrolysis efficiency [135].

According to a recent study, microwave-assisted proteolysis of WPs produced extensively hydrolyzed protein hydrolysates that did not trigger allergic reactions, unlike those generated through conventional heating methods [140]. From an industrial perspective, microwave processing offers significant advantages for liquid food products, including enhanced system flexibility, reduced thermal inertia, and potential energy savings compared to traditional heating techniques. This makes microwave-assisted proteolysis a promising and scalable approach for industrial applications in the dairy sector [140] (Table 3).

### 3.6. Pulsed Electric Field (PEF) Technology

Another example of physical hydrolysis is PEF processing, an innovative technology that enables the non-thermal hydrolysis of food proteins. In this method, a high-intensity electric field is applied (≈50 kV/cm), causing structural changes in proteins and enhancing enzymatic reactions without the need for excessive heat. Although the effects of PEF on WPs are known to vary with electric field intensity, there are contradictions in the literature. Some studies have suggested that PEF treatment has no significant impact on proteins [178]. However, optimizing the electric field density and processing time can enhance the functional and structural properties of peptides in WPs.

Additionally, thermal pretreatment combined with PEF may promote protein unfolding [179]. In industrial applications, PEF’s advantages include its non-thermal nature, reduced processing time, and improved yield of BAPs, making it an efficient method for producing functional food ingredients, particularly for dairy products like whey [180]. However, optimizing the PEF process, including electrode design and pulse parameters, is crucial for maximizing its benefits while avoiding potential negative effects like electrolysis [181]. This technique holds promise for scaling up BAPs production in the dairy industry (Table 3).

### 3.7. High-Pressure Processing (HPP)

High-pressure processing (HPP) is an innovative, eco-friendly, and non-thermal method that involves the application of pressures ranging from 100 to 1000 MPa, with or without additional heat treatment [135,182]. High-pressure processing denatures proteins by applying pressure, leading to biochemical changes as the volume decreases. The denaturation and/or aggregation of proteins depends on the pressure, temperature, time, and protein characteristics (e.g., pH and composition) [7,130]. At high pressures, β-LG aggregates via disulfide bond exchanges, leading to increased particle size and conformational changes [130]. In contrast, low pressures (<120 MPa) cause partial unfolding of β-LG, resulting in particle shrinkage and an increase in sulfhydryl groups [130]. When used as a pretreatment, HPP enhances enzymatic hydrolysis by destabilizing whey proteins, facilitating active site formation and chemical interactions. For instance, hydrolysis with chymotrypsin, pepsin, and trypsin produces more hydrophobic peptides when β-LG is pretreated with high-pressure processing compared to untreated proteins [183]. The efficiency of hydrolysis is influenced by factors such as pressure, temperature, time, pH, ionic strength, substrate, and enzyme type [135,183]. To prevent reduced susceptibility to proteolysis, hydrolysis should begin immediately after high-pressure processing. When HPP is applied during hydrolysis, protein agglomeration is avoided, ensuring more efficient peptide production [183]. HPP enhances foaming properties by increasing protein adsorption and intermolecular interactions at a neutral pH. However, pressures above 300 MPa reduce foam stability by causing protein opening [7]. At pressures over 100 MPa, HHP induces denaturation of β-LG and α-LA, complicating fractionation. The effects on β-LG are reversible at pressures above 300 MPa [166]. In a study applying batch HPP (0.1, 400, and 600 MPa for 10 min at room temperature) to bovine whey, peptides QEAKDAFLGSF and WENGECAQKK were obtained from β-LG through tryptic hydrolysis, with the highest yield at 400 MPa. However, 600 MPa pressure decreased peptide yield. Additionally, HHP (400 MPa) resulted in longer and more hydrophobic peptides during chymotrypsin hydrolysis of β-LG [135]. The application of HPP has some limitations, including batch operation and high infrastructure costs, ranging from 0.6 to 4 million USD, which account for 75–80% of the initial investment [184]. HPP alone has limited effects on breaking covalent bonds and producing BAPs. Therefore, it is typically combined with enzymatic hydrolysis to denature proteins and enhance enzyme access to cleavage sites, improving the efficiency and yield of BAPs production [137] (Table 3).

### 3.8. Subcritical Water

Subcritical water, also known as pressurized hot water extraction, superheated water extraction, or pressurized liquid extraction [137], has been positioned as an efficient and sustainable alternative to obtain peptides from various protein sources. Subcritical water has gained attention due to its non-toxic, non-flammable nature and the absence of solvent residues after extraction (Table 3). The use of subcritical water hydrolysis on WPI has been documented in the literature. A hydrolysis degree of 12% was achieved after 17 min at 298 °C, with the highest amino acid production (57.4 mg/g) occurring at 300 °C for 40 min [185]. Unfortunately, no information about biological activities was provided.

### 3.9. Ohmic Heating

Ohmic heating (also known as joules heating, electrical resistance heating, direct resistance heating, and electro-conductive heating) is the heating technique where heat is produced internally in the food being processed owing to its natural electrical resistance. This technology offers faster, more uniform heating, preserving the nutritional value of food better than conventional methods [186] (Table 3). Alizadeh and Aliakbarlu [187] investigated the antioxidant capacity of whey concentrate fractions subjected to ohmic heating (50 Hz, 0–240 V, 5–15 s), ultrasound-assisted extraction (24 kHz, 400 W, 25 mm titanium probe, 5–15 min), and enzymatic hydrolysis (pepsin). Both pretreatments increased the degree of hydrolysis (except for the combination of 5 min ultrasound-assisted extraction and proteolysis), with the highest hydrolysis achieved with a 15 min ultrasound-assisted extraction and 15 s ohmic heating treatment. This result was attributed to the breaking of covalent bonds and protein denaturation by heat. Both ultrasound-assisted extraction and ohmic heating alone showed similar effects [187]. The effect of ohmic heating on the production of BAPs is still not fully understood, with limited studies available. Sweet whey processed at low electric field intensities (2–4 V cm^−1^) showed higher ACE-inhibitory activity compared to pasteurization (72–75 °C), but activity decreased at 5 V cm^−1^ [186]. A similar trend was observed for antioxidant activity, likely due to the release of bioactive compounds during ohmic heating, although peptide formation was not characterized.

## 4. Prediction of Biological Activities of WP-Derived BAPs by In Silico Approaches

**Figure 3:**
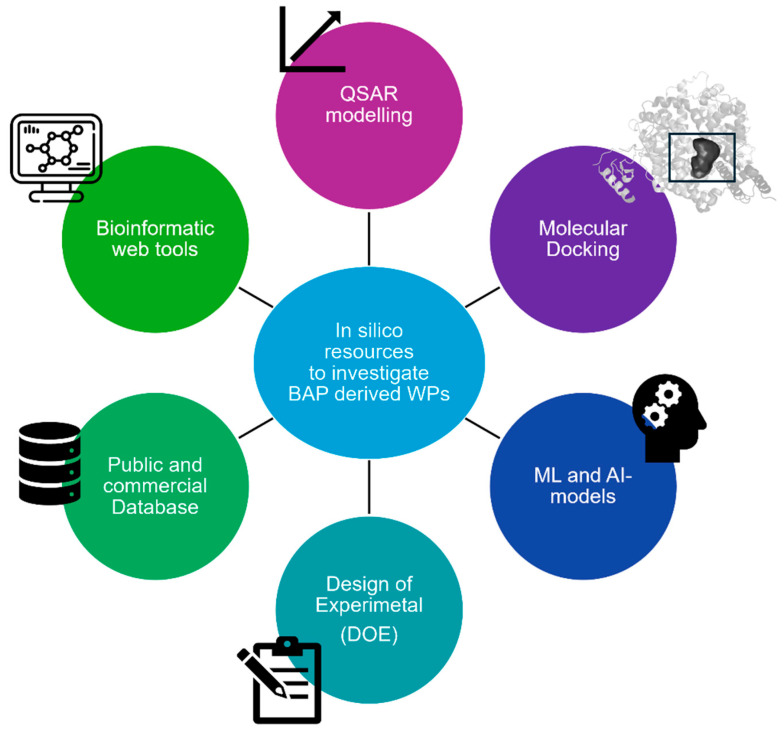
In silico tools to investigate BAPs derived from WP.

In the past, the study of BAPs derived from WPs was carried out based on traditional drug discovery approaches. Unlike drugs, BAPs are contained in complex food matrices, and identifying them among hundreds of peptides is very challenging. In this respect, the use of in silico methods has been gaining fertile ground to perform rational prioritization studies before in vitro screening and to enhance the success rate of the discovery of BAPs for food uses and possible medical applications by saving time and costs [31,187,188,189,190,191]. To this end, innovative in silico approaches are nowadays available, as depicted in Figure 3 and summarized in Table 4.

The discovery of BAPs can start by querying public and commercial databases of (i) WP-derived bioactive peptides (e.g., BIOPEP [192], AHTPDB [193], and milk bioactive peptide database (MBPDB) [194], just to mention a few), or (ii) pharmacological targets, such as metabolic enzymes (i.e., ACE, dipeptidyl peptidase-IV (DPP-IV), α-glucosidase), or (iii) markers of health and chronic diseases (i.e., cytokine levels, blood sugar, cholesterolemia, and sarcopenia). In this regard, a large number of collections dedicated to BAPs derived from WPs are nowadays freely available, as detailed in [31,195]. Notably, it emerges that a significant number of BAP-derived WPs are capable of exhibiting multitarget activities [31,190].

Nowadays, several web accessible platforms gather high-quality data from multiple publicly available sources in order to streamline the research of BAP discovery processes. For instance, experimental data are shared in the Open Pharmacological Concepts Triple Store (Open PHACTS) API (www.openphacts.org) based on a partnership involving academia, publishers, enterprises, and pharmaceutical companies with the aim of making drug discovery cheaper and faster. Similarly, bioinformatic platforms, such as ELIXIR, can be used for proteomic and peptidomic studies to predict the bioactivity, safety, and efficacy of BAPs, making research and data analysis more comprehensive [196]. Several works combining an in silico predictive tool for the enzymatic release of potent milk BAPs and in vitro studies [197,198,199,200] are worthy of mention.

A key role has been played by Quantitative Structure-Activity Relationship (QSAR) approaches. QSAR-based investigations have been carried out for the identification of WP-derived BAPs, which were successfully validated with experimental tests [191,201,202]. QSAR relies on mathematical models that correlate molecular and biological properties. Molecular descriptors particularly effective in elucidating the structure–activity relationship of BAPs are related to the global amino acid composition, as suggested by Dubchak et al. in 1995 [203]. The authors proposed as features the frequency of the property changes along the protein sequence and the distribution pattern of the property along the peptide sequence [20]. Next, the Amino Acid Index (AAI), Dipeptide Composition (DPC), tripeptide composition (TPC), and Grouped Dipeptide Composition (GDPC), as well as the structural features (e.g., β-sheet) and physicochemical peptide properties (e.g., hydrophobicity), are also considered as representative descriptors [195,204,205,206]. A QSAR study on whey-derived BAPs reported a correlation between hydrophobicity and positively charged amino acids in the C-terminal position and potent ACE-inhibitory activity. The correlation decreases for peptides with a length equal to or higher than six residues, thus highlighting the relevance of steric properties on ACE-inhibitory activity [207].

In the field of BAPs, molecular docking is a widely used in silico technique to predict the positioning and scoring of putative BAPs within the binding site of target proteins and to address the rational design of newer and more potent candidates [31,207,208,209].

For instance, molecular docking was successfully employed to identify bioactive oligopeptides effective toward ACE and to shed light on the rationale behind their binding [210,211]. In this respect, Norris et al. [210] discovered two new dipeptide stretches (i.e., Asp-Trp and Trp-Pro) with potent in vitro ACE-inhibitory properties. Furthermore, a recent docking study showed that 14 dipeptides from whey and other proteins inhibit ACE by engaging key binding site residues such as His353, Lys511, and Tyr520 through the formation of hydrogen bonds [212].

By exploiting docking simulations, Tondo et al. [157] assessed the activity of β-lactoglobulin-identified BAPs for hypertensive properties based on ACE inhibition. In a parallel investigation, Gambacorta et al. [213] proved that some of these peptides (i.e., IIAEKTK, IPAVF, and MHIRL) could be effective in interfering with SARS-CoV-2 spike main proteases. More specifically, the docking study revealed the potential affinity of these β-lactoglobulin-derived peptides toward the Human Rhinovirus 3C protease of SARS-CoV-2. These three peptides confirmed the importance of the hydrogen bond network within the binding sites, involving residues Q189 and N142, promoting conformational changes for a more effective binding.

A molecular docking approach can also help to unveil latent similarities among peptides and protein targets apparently uncorrelated. For instance, ACE and DPP-IV share similar mechanisms of inhibition and common structural features: both enzymes are able to hydrolyze terminal dipeptides based on their catalytic activity, and their cleaving actions are preferably addressed toward proline residues. Based on this, some BAPs showed their ability to inhibit ACE and DPP-IV in a similar way, as reported in a recent work by Gu et al. [214]. Several whey-derived peptides, such as RGP, FPK, KFTW, and KPW, have been reported to inhibit ACE and DPP-IV. Notably, RGP and KPW form hydrogen bonds with key ACE residues (i.e., Ala354, His383, Gln281, Glu384, and Glu162); otherwise, RGP, FPK, KFTW, and KPW engage a critical hydrogen bond with Arg358 of DPP-IV. Additionally, three out of these peptides (FPK, KFTW, and KPW) engage with only a hydrophobic residue in DPP-IV—that is, Phe357 [214]. As DPP-IV cleaves dipeptides with a proline residue at the penultimate position, DPP-IV inhibitory activity for peptides is expected with Xaa-Pro or Xaa-Pro-Yaa motif [215]. The simulated DPP-IV hydrolysates of KPW and FPK are KP and FP, respectively, reported to exhibit strong DPP-IV-inhibitory activity [214].

One of the major challenges with WPs is flocculation and precipitation. Complexes with sodium alginate can help mitigate these critical issues and stabilize WPI. Molecular docking integrated with deep learning models highlighted specific intermolecular interactions between whey peptides and sodium alginate [216].

The use of Artificial Intelligence (AI) and machine learning (ML) significantly changed the perspective for the rational design and optimization of BAP, including those deriving from [195,217]. ML models have been proven to identify common features and biological functions from large datasets of BAPs generated through enzymatic hydrolysis. Among the ML algorithms, Random Forest (RF), Support Vector Machines (SVMs), K-Nearest Neighbor (KNN), Naive Bayes (NB), Artificial Neural Networks (ANNs), and Logistic Regression (LR) have been exploited to identify and characterize whey-derived BAPs, as well as to profile their functions [195]. For instance, Morales García et al. [218] identified 2572 peptides under different conditions and with various types of enzymes by using different ML algorithms. Among these, 499 peptides were found to have potential antioxidant activity.

Moreover, ML can be applied in various ways to optimize whey management by enhancing the efficiency of whey separation from the curd. For instance, a recent approach suggested by Jox et al. [219] exploits both time-series data (e.g., fermentation and storage parameters) and non-time-series data (e.g., documentation and turbidity measurements) to train eleven models based on different ML algorithms. This protocol utilized five out of six steps of the CRISP-DM (CRoss-Industry Standard Process for Data Mining) methodology, which was specifically adapted to address whey management. BAPs obtained after hydrolysis needed to be separated using ElectroDialysis with Filtration Membranes (EDFM), which operate based on molecular mass exclusion and charge selectivity. Sanchez-Reinoso et al. [220] derived ML models based on DT and Binary Greedy Networks to estimate the peptide migration rate in EDFM by incorporating membrane fraction characteristics and peptide features.

AI-based algorithms are also widely implemented in several bioinformatic tools aiming to predict secondary and tertiary peptide structures from amino acid sequences (e.g., PEP-FOLD PepLook or AlphaFold 2) or function (e.g., APPTEST). Jia et al. exploited a peptide sequence-based multi-label deep learning approach to discover novel whey-derived ACE inhibitors [221]. On the other hand, SPRINT-STR uses RF algorithms to predict various protein-peptide complexes [222]. AI- and ML-based tools could also be exploited for the prediction and characterization of peptide binding sites [223,224,225]. An example is given by Baba et colleagues by using PepSite2 to predict peptide-binding sites on human ACE and renin based on the ACE inhibition of 185 peptides derived from 27 hydrolysates [212]. Additionally, the integration of ML models with docking represents a further option to consolidate the goodness of results. For instance, Petra et al. recently presented a novel pipeline for the screening of proteomes representative of complex protein-rich foods, including WPs. In this work, ML models classified the peptide functional groups from the whey proteome of colostrum from indigenous Greek goats, while molecular docking was employed to assess the binding affinity of the proposed ACE and DPP-IV inhibitory peptides [226].

Last but not least, the Design Of Experiments (DOE) in combination with Response Surface Methodology (RSM) represents an in silico workflow currently employed to aid the optimization of BAPs release during enzymatic hydrolysis and the fermentation of WPs [227]. The aim is to find the optimal number of experimental tests returning the maximum information content.

All DOE protocols consider hierarchical steps as follows: (i) definition of the scope/aims to be achieved from given experiments; (ii) define the biological effect/activity output to be optimize (e.g., antioxidant effects or DPP-IV or ACE potential activity); (iii) appropriate tuning of parameters (e.g., temperature, incubation time, and pH, just to mention a few) influencing the output of interest; (iv) selection of the experimental design, taking into account the boundaries of the study (e.g., the range of pH or temperature in which the enzyme is active). By considering the DOE protocol, the data obtained by experimental test(s) will be used to develop a mathematical model by using the Multiple Linear Regression (MLR) algorithm, returning a solid relationship between the effect of the parameters and the biological effect or activity to be optimized. Finally, the RSM analysis consists of a collection of mathematical and statistical techniques based on the fit of a polynomial equation to experimental data [227,228]. In dairy peptide research, DOE has been employed to optimize the release of BAPs with biological activity [227,229,230,231]. Table 4 provides a comparative overview of in silico techniques used for the discovery of bioactive peptides derived from whey proteins (WP-derived BAPs).

## 5. Regulatory Frameworks and Safety Assessments for WP-Derived BAPs: Global Perspectives and Challenges

WPs and WPHs serve as a valuable source of BAPs [15,232] and, due to their technological properties, are widely used as ingredients or in the production of functional foods with specific characteristics, providing efficient solutions to meet protein and nutritional needs while supporting overall health [46]. Over the years, various products have indeed been marketed, such as baked goods, extruded products, dairy products, beverages, and others [232]. The use of WPs in food products is subject to regulatory requirements and labeling regulations: while whey-fortified food products offer several nutritional benefits, there are also challenges and limitations, such as the regulation and approval of protein fractions or peptides, whose benefits require further confirmation.

Some WPIs have been granted Generally Recognized As Safe (GRAS) status and are authorized for marketing in the United States [233,234].

Several bovine LF (bLF) products have received GRAS status from the US FDA [235,236,237,238,239,240], and bLF is permitted for use in infant formulae and conventional foods in Japan, China, Korea, and Taiwan. EFSA assessed the safety of bLF in 2012 for various food categories, concluding that the proposed intake levels were safe. In contrast, EFSA has not previously evaluated the safety of bovine LP (bLP), which is accepted in dietary supplements in the USA and Japan [241] and as a processing aid in New Zealand [242].

In Europe, the regulatory framework governing the labeling of WPs is primarily defined by Regulation (EU) No. 1169/2011 on food information to consumers, along with specific provisions under food safety and novel food regulations such as Regulation (EU) 2015/2283.

In 2018, the EFSA Panel on Nutrition, Novel Foods and Food Allergens (NDA) concluded that whey basic protein isolate from skimmed cow’s milk is safe for human consumption under the proposed conditions of use as infant and follow-on formula, meal replacement beverages, foods for special medical purposes, and food supplements [243].

The Novel Food (NF) consists of basic WPs obtained via ion exchange chromatography from skimmed cow’s milk. Its main components—bLF, LP, and transforming growth factor b2 (TGF b2)—share 70%, 83%, and 99% amino acid sequence homology, respectively, with their human milk counterparts. The highest estimated intake is 24.8 mg/kg bw/day in infants and 27.8 mg/kg bw/day in toddlers [242]. The composition, production process, and stability data raise no safety concerns, and the NF is not nutritionally disadvantageous at the proposed intake levels. There are no concerns regarding genotoxicity, with a No Observed Adverse Effect Level (NOAEL) of 2000 mg/kg bw/day in a subchronic rat study (highest dose tested) [244,245,246]. The margin of exposure (MOE) is 154 for adults, while for infants and toddlers, it is 81 and 72, respectively. Given that bLF, bLP, and TGF-β2 are already present in bovine-based infant formula, their increased intake from the NF is not considered a risk for the EFSA NDA Panel [247].

In 2019, following a request from the European Commission (EC), the EFSA NDA Panel was asked to deliver a scientific opinion on whey basic protein isolate for extended uses in foods for special medical purposes and food supplements for infants as a novel food (NF) pursuant to Regulation (EU) 2015/2283 [246]. The applicant requested an extension of the conditions of use to include infant formula (30 mg/100 g in powder form and 3.9 mg/100 mL when reconstituted) and follow-on formula (30 mg/100 g in powder form and 4.2 mg/100 mL when reconstituted) as food for special medical purposes, as well as its inclusion in food supplements for infants (25 mg/day). The Panel considered that the proposed extended uses would not lead to an increased intake of the novel food compared to the levels assessed in its 2018 opinion. Consequently, the Panel concluded that whey basic protein isolate is safe for the proposed extended uses, including its incorporation into infant and follow-on formulae, total diet replacements, foods for special medical purposes, and food supplements [246].

In recent years, the interest of the EC has been increasingly shifting toward hydrolysates derived from sources of skimmed cow’s milk, and since 2020, several scientific opinions on the topic have been requested from the EFSA [248,249,250,251,252].

Overall, whey-peptide-based products addressing consumption must meet the requirements of the EC 1924/2006 regulation with health claims [253]. The incorporation of WPHs in infant formula formulations is regulated by [254,255].

According to the scientific opinion published in 2025, to assess the nutritional safety and suitability of a protein hydrolysate derived from skimmed cow’s milk [248], the EFSA found the hydrolysate to be sufficiently characterized and evaluated an intervention study where an infant formula containing 2.3 g/100 kcal of this protein hydrolysate, consumed exclusively for three months, supported similar growth to a formula based on intact cow’s milk protein with 1.9 g/100 kcal. No concerns were raised regarding adverse events or formula tolerance. The panel determined that the protein hydrolysate is a nutritionally safe and suitable protein source for both infant and follow-on formula, provided the formula contains at least 2.3 g/100 kcal of protein and complies with Regulation (EU) 2016/127 and the amino acid profile specified in Annex IIIA [252].

The Codex Alimentarius provides a standard for whey powders, specifically outlined in CXS 289-1995 (formerly CODEX STAN A-15-1995) [256]. This standard covers whey powder and acid whey powder, detailing their composition, permissible additives, hygiene requirements, and labeling provisions. It is applicable to products intended for direct consumption or further processing. However, whey protein hydrolysates are not specifically addressed within the current Codex standards. This absence indicates a regulatory gap concerning the classification, safety assessment, and labeling of these hydrolyzed products. Recognizing this gap, AOAC International has initiated efforts to develop voluntary consensus standards for methods used to characterize WPHs. These standards aim to support the development and validation of official analytical methods, which could potentially be adopted by Codex Alimentarius in the future.

## 6. Conclusions and Future Perspectives

WPs have a high biological value largely determined by their content of essential amino acids and BAPs. The research of BAPs pushed WPs to the forefront of the functional food sector by making the development of novel technology of WPs recovery and transformation into BAPs an urgent challenge to be addressed; although several efforts have been undertaken in this direction, WPs processing and emergent derivatives remain crucial due to the low yields and time cost and environmental impacts.

Green technologies such as HPP, microwave, ultrasound, and PEF, coupled with enzymatic hydrolysis, seem to overcome these limitations; these technologies have been found to reduce the time and costs of processing and improve the yield of BAPs. In addition, the achievement of WP hydrolysis in reactors should contribute to increased efficiency and production yields and improve production stability between batches; the employment of membranes or immobilized enzyme reactors would save costs associated with enzymes by reducing processing costs. Although this review highlights multiple lines of evidence, significant progress is still needed in this field.

These techniques, indeed, selected for their sustainability and industrial scalability, will increasingly need to be implemented by high-throughput screening, AI-driven bioinformatics, and multi-omics (proteomics and metabolomics) that can improve the identification and characterization of BAPs.

By saving time and reducing costs, in silico approaches can accelerate the identification of BAPs from complex mixtures and facilitate the study of protein–peptide interactions by predicting their biological affinity; these computational methods have been found to be effective in safeguarding the sustainability of the discovery process. This approach can be used to improve the delivery of active peptides through more appropriate encapsulation methodologies, leading to tailored nutritional solutions for different populations, such as athletes, elderly individuals, or people with metabolic disorders. It is therefore evident that there is a strong interest in developing new algorithms and mathematical models to address these concerns. However, here too, there are several limitations; among the latter, the validation studies represent the bottleneck for consolidating strategies that improve peptide manufacturing processes.

With growing consumer demand for high-protein and functional foods, the WP and BAP market is projected to expand significantly in the near future. The growing interest in biologically active whey-derived peptides calls for clearer regulations regarding their use in the food sector, particularly in terms of safety and risk assessment. Future research should also focus on standardization studies and methods to strengthen the stability of the WPs derivatives. An improvement in shelf life can be ensured through the implementation of good manufacturing practices and rigorous quality control. Recent legislative updates acknowledge the high nutritional value of WPs-based products and encourage manufacturers to pursue this direction.

## Figures and Tables

**Table 2:** Major and minor whey proteins (WPs) in bovine milk and related properties.

Protein	Concentration (g/L)	Molecular Weight (kDa)	Recovery Process	Biological Functions	References
*β*-lactoglobulin (*β-LG*)	3.2–3.6	18.277	Ultrafiltration and ion exchange (diethylaminoethyl cellulose)	Transporter (Retinol, Palmitate, fatty acids, Vitamin D). Regulator in the mammary gland to carry out phosphorus metabolism. Synthesis of glutathione stimulation	[61,62,66]
*α-la*ctoalbumin (*α-LA*)	1.2–1.6	14.175	Ultrafiltration, precipitation, and ion exchange (sepharoseiminodiacetate-Cu^2+^)	Prevention of cancer; lactose synthesis. Treatment of chronic stress-induced disease	[66,70,71,72,73,74,75]
Albumin of the bovine serum (BSA)	0.4–0.5	66.267	Ultrafiltration, precipitation, and ion exchange (MAG polyethyleneimine anion exchange)	Transport of low-molecular-weight fatty acids; prevention of cancer; immunomodulation: disease protection through passive immunity	[78,79]
Immunoglobulins (IGs)	0.7–1.0	25 (light chain) 50–70 (heavy chain)	Ion exchange (sepharose 6B-organic ligands Cu^2+^)	Antimicrobial and antiviral activity. Lower cholesterol and blood pressure	[85]
Glycomacropeptide (GMP)	1.2–1.3	6700	Ultrafiltration with some modifications (membrane 5 kDa) Ultrafiltration combined with ion-exchange chromatography	Effect over phenylketonuria illness Antibacterial, prebiotic, remineralizing activity; Modulator of digestion and metabolism; anti-tumoral; immuno-modulation	[87]
Lactoferrin (LF)	0.1–0.2	80	Hydrophobic interaction. Affinity separation (superparamagnetic polyglycidyl). Methacrylate particles coupled with heparin. Ion exchange	Biological effect as anti-inflammatory, antifungal, antiviral, antibacterial, antibiofilm, and anticancer	[12,13,66,101]
Proteose–peptone (Pp)	0.6–1.8	28	Hydrophobic interaction and chromatography with a dual salt system	Action as bifidogenic factor	[118,130,131]
Lactoperoxidase (LP)	Traces	70	Ion exchange (sepharose 6B-reactive red 4 dye and cryogel embedded with cellulose beads)	Association with 17 antimicrobial activities	[122,132,133]

**Table 3:** Benefits and limitations of conventional and novel technologies in industrial production of bioactive peptides (BAPs) from whey proteins (WPs).

Methodology	Advantages	Disadvantages	Industrial Scalability	Estimated Cost	References
Microbial fermentation	Efficient and economical method; utilizes natural microbial enzymes	Low yield; lacks specificness of peptide generation; potential consumption of peptides by microbes; batch variability; time-consuming	Moderate to high—used in functional dairy products and supplements	Medium	[141,142,143,144,145,146,147,148,149,150,151,152]
Enzymatic hydrolysis	Reliable; efficient; high specificity and selectivity; mantain nutritional value	Expensive due the high cost of enzymes and the additional costs for processes optimization	High—widely used in food, pharma, nutraceuticals	Medium to high	[153,154,155,156,157,158,159,160,161,162,163]
Chemical hydrolysis: Acid hydrolysis	Complete protein breakdown; simple reagents (such as HCl)	Destroys some amino acids (such as tryptophan); harsh conditions	Low to moderate—not ideal for food applications	Low to medium	[164,165,166,167]
Chemical hydrolysis: Alkaline hydrolysis	Effective at high protein solubilization	Causes racemization and lysin or alanine formation; Alters nutritional value	Low—rarely used in food due to safety concerns	Low	
Heat treatment	Simple and cost-effective; Promotes protein unfolding and digestibility; may enhance BAPs release when combined with enzymes	Non-specific; may degrade sensitive amino acids; can denature proteins to expose peptide bonds to hydrolysis	High—widely used in food industry	Low to medium	[168,169]
Ultrasound treatment	Faster start-up; extraction selectivity; high process control. Eco-friendly (reduced temperature and time, and faster mass and energy transfer). Preserves nutritional quality	Alone cannot break peptide bonds; mainly employed as pretreatment; potential alteration of protein structure. Requires optimization for different substrates	Moderate—used in liquid processing	Medium	[42,135,138,170,171,172,173,174,175,176]
Microwave treatment	Fast heating and energy-efficient. Enhances enzymatic hydrolysis efficiency. Economic sustainability. Ease of integration, and manageable processing conditions	Equipment costs. Used as pretreatment can favor changes in protease cleavage sites	Moderate—requires adapted equipment	Medium	[135,140,177]
Pulsed electric field technology	Non-thermal; enhances cell permeability; short treatment times	Limited data on peptide extraction; high initial investment	Low to moderate—emerging technology	High	[178,179,180,181]
High pressure processing	Omogeneous and constant pressurization at ambient temperatures. Moderate energy cost. Reduced processing times. Non-thermal; preserves functional properties. Can alter protein structures to enhance hydrolysis	High equipment costs. Limited penetration for dense systems	High—used in premium and functional food sectors	Medium	[130,135,137,166,182,183,184]
Subcritical water	Green solvent. Can hydrolyze proteins without chemicals	Requires high-pressure equipment; potential degradation of heat-sensitive peptides	Low—mostly at research/pilot scale	High	[137,185]
Ohming heating	Uniform and rapid heating. Energy-efficient	Limited research on peptide extraction. Potential equipment corrosion	Moderate—needs scale-up optimization	Medium	[186,187]

**Table 4:** Comparative overview of in silico techniques for WP-derived BAP discovery.

In Silico Technique	Purpose/Application	Advantages	Limitations	Estimated Cost	Efficiency/Notes
Database Mining (e.g., BIOPEP, MBPDB)	Identification of known BAPs from WPs	Rapid access to curated sequence peptide data	Limited to known peptides; no novel activity prediction	Free/Low	High for initial screening and hypothesis of peptide design
QSAR Models	Predict bioactivity from peptide structure	High throughput; interpretable; cost-effective	Requires quality training data; limited generalizability	Medium	Effective when trained on relevant peptide data
Molecular Docking	Predict binding affinity to targets (e.g., ACE, DPP-IV)	Reveals peptide-target interactions; supports rational design	Results can be static and may not reflect in vivo dynamics	Medium	Useful for mechanistic insight and virtual screening prioritization studies
ML/AI Algorithms	Predict peptide functions (e.g., antioxidant, ACE inhibition)	Handles large datasets; uncovers complex patterns	Requires robust datasets; may lack interpretability	Varies (often free)	High accuracy with sufficient training data; adaptable to different applications
EDFM + ML Models	Predict peptide behavior during membrane-based separation	Combines chemical and process engineering data	Needs experimental calibration and complex feature sets	Medium/High	High accuracy in recovery/purification modeling with proper data integration
Peptide Structure Prediction tools	Predict 2D/3D peptide conformations	Provides structural insight; aids docking and SAR studies	Computationally intensive; accuracy varies by method	Free/Low	High predictive power
DOE + RSM	Optimize peptide release during hydrolysis/fermentation	Reduces experimental runs; statistically rigorous	May oversimplify biological systems	Medium	High efficiency for experimental design; widely used in food science
